# Artificial Intelligence in Autism Spectrum Disorder Diagnosis: A Scoping Review of Face, Voice, and Text Analysis Methods

**DOI:** 10.1002/hsr2.71476

**Published:** 2025-11-17

**Authors:** Fatemeh Mohammadi, Hassan Shahrokhi, Afsoon Asadzadeh, Saeed Pirmoradi, Ali Moghtader, Peyman Rezaei‐Hachesu

**Affiliations:** ^1^ Department of Health Information Technology, School of Management and Medical Informatics Tabriz University of Medical Sciences Tabriz East Azerbaijan Iran; ^2^ Autism and Related Neurodevelopmental Disorders Research Team Tabriz University of Medical Sciences Tabriz East Azerbaijan Iran; ^3^ Department of Psychiatry Tabriz University of Medical Sciences Tabriz East Azerbaijan Iran; ^4^ Clinical Research Development Unit of Tabriz Valiasr Hospital Tabriz University of Medical Sciences Tabriz East Azerbaijan Iran; ^5^ Faculty of Electrical and Computer Engineering University of Tabriz Tabriz Iran; ^6^ Emergency & Trauma Care Research Center Tabriz University of Medical Sciences Tabriz East Azerbaijan Iran

**Keywords:** artificial intelligence, autism, diagnosis, facial images, text analysis, voice

## Abstract

**Background:**

Autism is a complex neurodevelopmental condition affecting social interaction and behavior. Traditional diagnostic methods, relying on observational techniques and interviews conducted by trained professionals, remain the gold standard for ASD diagnosis. However, these methods can be time‐consuming and may be influenced by subjective factors. Recent advancements in artificial intelligence (AI) offer promising approaches to augment existing methods, potentially enhancing efficiency and providing additional objective data through facial, vocal, and textual analysis.

**Objective:**

The objective of this study was to conduct a comprehensive review of artificial intelligence applications in autism spectrum disorder (ASD) diagnosis, specifically focusing on facial, vocal, and textual analysis methods.

**Methods:**

A comprehensive search was conducted in PubMed, Web of Science, Scopus, and Google Scholar. The findings were reported in accordance with the PRISMA checklist. Data were collated and summarized, and results were reported qualitatively, adopting a narrative synthesis approach.

**Results:**

In facial image analysis, deep learning algorithms demonstrated high accuracy in identifying autism‐related facial features, algorithms such as Xception achieved 98% accuracy, while hybrid approaches like the combination of Random Forest (RF) and VGG16‐MobileNet showed accuracy at 99%. Voice analysis studies utilized both traditional machine learning methods and advanced deep learning techniques, achieving accuracies between 70% and 98% in detecting atypical speech patterns and prosodic abnormalities associated with autism. Text‐based analyses showed potential in identifying linguistic markers of autism through natural language processing techniques. Overall, Deep learning approaches were mainly employed in facial image analysis for autism diagnosis. In contrast, voice and text recognition studies utilized machine learning algorithms.

**Conclusions:**

This review demonstrates artificial intelligence's significant role in diagnosing autism spectrum disorder (ASD). These AI‐driven approaches can complement traditional diagnostic methods, potentially leading to earlier interventions and improved outcomes for individuals with ASD.

**Abbreviations**
ADI‐R: autism diagnostic interview‐revised
ADOS‐2: autism diagnostic observation schedule 2nd edition
ASD: Autism spectrum disorder
BERT: Bidirectional encoder representations from transformers
CARS: childhood autism rating scale
CNNs: Convolutional neural networks
MLP: multilayer perceptron
PNN: probabilistic neural network
Q‐CHAT: The quantitative checklist for autism in toddlers

## 1 Introduction

Autism spectrum disorder (ASD) is a neurodevelopmental [1] disorder characterized by social and communicative deficits and repetitive behaviors emerging at ages 1 to 4 years [2] marked by deficits in social interaction, language and communication impairments, repetitive and stereotypical behaviors, limited interests, and activities, and occasionally heightened sensitivities to sensory stimuli such as sound or touch, along with potential displays of aggression [3]. This rate varies by sex assigned at birth, with the prevalence in males 3.8 times higher than in females [4]. According to updated data, ASD global prevalence is about 119 per 10,000 [5].

Early identification of ASD is particularly difficult, for the following reasons: (1) the range of what is considered typical, healthy social‐communication development is very wide; (2) certain ASD symptoms such as language patterns cannot be detected and evaluated before the child reaches the age of 2 years; (3) diagnosis depends on clinical expertise rather than on a biological marker; (4) early signs of ASD and other neurodevelopmental disorders partly overlap leading to complex diagnostic issues; and (5) ASD screening tools show moderate sensitivity and specificity [6].

In the domain of psychiatry and psychology, however, medical decision‐making relies nearly exclusively on observational or paper‐and‐pencil instruments [7]. The primary and conventional method used by experts is interview‐based, where the condition of the patients is assessed by the different questionnaire protocols such as autism diagnostic observation schedule 2nd edition (ADOS‐2) [8], autism diagnostic interview‐revised (ADI‐R) [9], childhood autism rating scale (CARS) [10], The Quantitative Checklist for Autism in Toddlers (Q‐CHAT) [11]. The main flaw of these traditional methods is biasness, such as the physician's competence, skill, and timetable. In addition, the patient's parents or attendant cannot always give accurate data or fill out the questionnaire forms correctly. All these factors can influence the accuracy of interview‐based ASD diagnosis [12]. ASD's genetic heterogeneity presents a key challenge: how diverse biological causes lead to shared behavioral traits. While specific mechanisms may vary, all disrupt normative developmental processes, creating a common divergence from typical social development. This shared divergence underlies the spectrum of ASD phenotypes. These behavioral differences manifest in quantifiable patterns of social attention, vocal prosody, and sensory processing. These conserved traits shape later social specialization, and their disruption in ASD creates measurable behavioral differences [13].

To address these limitations, modern approaches incorporate technological tools to assist in diagnosing and monitoring treatment progress. From brain imaging technologies to blood and genetic tests, instruments that assist medical decision‐makers [7]. One of the technologies includes sensor technologies such as wearable devices [14], virtual reality [15], robotics [16], and artificial intelligence [17]. Recent advances in computer vision and artificial intelligence are poised to rapidly advance research and clinical decision‐making in psychiatry by introducing reliable and granular tools within a new paradigm: computational behavior analysis [7]. AI has been used for symptom recognition, classification, diagnosis, and outcome. By capturing subtle behavioral patterns imperceptible to human observers, AI reduces diagnostic subjectivity while improving accuracy [18].

Numerous studies have been conducted to explore significant features of autism in various ways through artificial intelligence, such as eye tracking, facial recognition, medical image analysis, voice recognition, and text processing [19, 20, 21, 22, 23]. For instance, In the field of autism diagnosing through the face with artificial intelligence, various studies have been conducted, for example, a study by Bakri et al. (2023) [24], Masahito et al. (2020) [25], and Shafiul Alam et al. (2022) [12] has shown that Facial expression analysis is critical in understanding the non‐verbal communication deficits often seen in children with ASD. Recent studies have shown a high level of accuracy in the diagnosis of autism using facial image analysis based on artificial intelligence. Some studies have reported an accuracy rate of 98%, 99% in the classification of ASD cases [24, 26].

In addition to analyzing facial features, several studies have demonstrated the utility of artificial intelligence in assessing voice and text data. For instance, Mohanta et al. (2022) [27] and Mukherjee et al. (2023) [19] highlight how AI can effectively contribute to the diagnosis and understanding of autism through various modalities, enhancing the overall diagnostic process.

Given the prevalence and complexity of autism diagnosis, many studies have investigated AI‐based approaches. However, our research has uncovered a notable absence of a thorough review that synthesizes findings across these various modalities. This review aims to fill that gap by providing a holistic overview of the current state of AI in ASD diagnosis. The significance of this study lies in its potential to offer psychiatrists and psychologists a deeper understanding of AI features relevant to ASD diagnosis, guide AI developers in selecting suitable approaches and algorithms for ASD applications, and help researchers illuminate underexplored areas for future research. The primary goal of this study is to conduct a comprehensive review of artificial intelligence applications in autism spectrum disorder (ASD) diagnosis, specifically focusing on facial, vocal, and textual analysis methods.

## 2 Methods

This study is part of the ABBILAR project. The ABBILAR project is a research initiative at Tabriz University of Medical Sciences in the field of comprehensive management of ASD at the individual, family and social levels [28].

### 2.1 Databases and Search Strategy

The reporting of this scoping review was guided by the standards of the preferred reporting items for systematic reviews and meta‐analyses extension for scoping reviews (PRISMA). The PRISMA‐ScR checklist is available in Supplementary Information 1. The search was conducted using PubMed, Web of Science and Scopus on February 19, 2024, without any time limit. To identify additional potential research papers, we have also randomly searched and selected some of the recent studies from Google Scholar, which were searched using the keywords [(artificial intelligence) AND (autism OR ASD) AND (diagnosis) AND (face OR voice OR text)] in all papers, for example, the search strategy of one of the databases is available in Supplementary Information 2. All searches were restricted to articles written in English and published in peer‐reviewed journals. The inclusion criteria are described in Table 1. The two authors screened the identified studies for inclusion in the review. Discrepancies were then resolved through discussion and by consulting the third author when necessary. Then, the full text of the relevant records was obtained and checked against the criteria for final inclusion in the review.

**Table 1:** Inclusion and exclusion criteria for this review.

Inclusion criteria	Exclusion criteria
Children with autism	No full‐text available
Articles that were published in English	Full text in languages other than English
Articles that used face, voice, or text for recognition	Articles on the classification of autism for adults
Articles that used artificial intelligence as a diagnostic tool	Articles that were retracted
Studies of children (2–18 y) with ASD, Children with autism	Articles that did not use artificial intelligence for diagnosis
……	Adult populations (> 18 y)

### 2.2 Study Selection

The records resulting from the electronic search were imported into an EndNote X6 library, and the duplicates were removed. Following the removal of 119 duplicates, a primary inspection of study titles and abstracts was conducted. Following this initial screen, full‐text articles of the remaining studies were retrieved and assessed against eligibility criteria. Records were then independently reviewed by two experts in the field, and all reviewers met to resolve any conflicts and to ensure that selected papers were in line with the aims of the review. Studies were synthesized and categorized according to their main area of focus, and the findings were presented narratively to provide a summary related to the research questions.

### 2.3 Data Extraction

We will use EndNote to collect and upload all identified citations. Duplicates will be removed. The papers were searched according to the inclusion criteria in three steps, including title screening, abstract screening, and full‐text screening. Titles and abstracts of the papers of current interest will then be assessed independently by 2 reviewers against the inclusion and exclusion criteria listed above. After selecting the related papers, the following data were extracted: authors & year, title, country, Importance of work, sample size, age, statistical analysis or validation, main results, authors' Conclusions, and users. The extracted tables are described in Tables 1, 2, 3 (Supplementary Information 3).

**Table 2:** Technical aspects for facial images.

Title (Reference)	Learning approach	AI algorithm (accuracy)	Other statistical analysis
The Early Detection of Autism Within Children Through Facial Recognition: A Deep Transfer Learning Approach [29]	Deep Learning	MobileNet [89] VGG16 [82] ResNet50 [69]	precision: 85 recall: 91 precision: 80 recall: 85 AUC: 75
Improved Transfer‐Learning‐Based Facial Recognition Framework to Detect Autistic Children at an Early Stage [30]	Machine Learning Deep Learning	AdaBoost [66] Decision Trees [66] Gradient Boosting [73] K‐Nearest Neighbors [68] Logistic Regression [69] Multi‐Layer Perceptron Perceptron [67] Naive Bayes [68] Random Forest [76] Support Vector Machine [74] XGBoost [74] DenseNet121 [83] ResNet50 [81] VGG16 [76] VGG19 [71] MobileNet‐V1 [90] MobileNet‐V2 [64]	Sensitivity: 66 Specificity: 66 Sensitivity: 66 Specificity: 66 Sensitivity: 73 Specificity: 73 Sensitivity: 68 Specificity: 68 Sensitivity: 69 Specificity: 69 Sensitivity: 67 Specificity: 67 Sensitivity: 68 Specificity: 68 Sensitivity: 76 Specificity: 76 Sensitivity: 74 Specificity: 74 Sensitivity: 74 Specificity: 74 Sensitivity: 83 Specificity: 83 Sensitivity: 81 Specificity: 81 Sensitivity: 76 Specificity: 76 Sensitivity: 71 Specificity: 71 Sensitivity: 90 Specificity: 90 Sensitivity: 64 Specificity: 64
Identification of Autism in Children Using Static Facial Features and Deep Neural Networks [31]	Deep Learning	MobileNet (NA) Xception (NA) EfficientNetB0 (NA) EfficientNetB1 (NA) EfficientNetB2 (NA)	Sensitivity: 86 Specificity: 83 AUC: 92 Sensitivity: 88 Specificity: 91 AUC: 96 Sensitivity: 84 Specificity: 88 AUC: 93 Sensitivity: 86 Specificity: 94 AUC: 95 Sensitivity: 84 Specificity: 93 AUC: 94
Hybrid Techniques of Facial Feature Image Analysis for Early Detection of Autism Spectrum Disorder Based on Combined CNN Features [24]	Hybrid	XGBoost & VGG16‐ResNet101 [98] XGBoost & ResNet101‐MobileNet [97] XGBoost & VGG16‐MobileNet [98] RF & VGG16‐ResNet10 [97] RF & ResNet101‐MobileNet [98] RF & VGG16‐MobileNet [99]	Precision: 95 Sensitivity: 96 Specificity: 95 Precision: 94 Sensitivity: 94 Specificity: 94 Precision: 95 Sensitivity: 95 Specificity: 95 Precision: 94 Sensitivity: 95 Specificity: 95 Precision: 95 Sensitivity: 95 Specificity: 95 Precision: 95 Sensitivity: 95 Specificity: 96
Empirical Study of Autism Spectrum Disorder Diagnosis Using Facial Images by Improved Transfer Learning Approach [12]	Deep Learning	VGG19 [86] Xception [92] ResNet50V2 [90] MobileNetV2 [86] EfficientNetB0 [85]	precision: 86 recall: 86 precision: 90 recall: 90 precision: 90 recall: 90 precision: 86 recall: 86 precision: 85 recall: 85
Efficient Deep Learning‐Based Data‐Centric Approach for Autism Spectrum Disorder Diagnosis from Facial Images Using Explainable AI [26]	Deep Learning	MobileNetV2 [97] ResNet50V2 [97] Xception [94]	precision: 97 recall: 97 precision: 97 recall: 97 precision: 97 recall: 94
Detection of autism spectrum disorder using transfer learning [32]	Deep Learning	VGG19 [29] ResNET50 [52] InceptionV3 [80] NASNetLarge [86]	precision: 25 recall: 50 precision: 25 recall: 50 precision: 48 recall: 48 precision: 42 recall: 42
Deep Learning Algorithms to Identify Autism Spectrum Disorder in Children‐Based Facial Landmarks [33]	Deep Learning Hybrid	MobileNet‐V1 (92) VGG‐16 with ML: logistic regression [82] VGG‐16 with LinearSVC(81) VGG‐16 with RF [78] VGG‐16 with decision tree [66] VGG‐16 with gradient boosting [75] VGG‐16 with KNN [52] VGG‐16 with MLPClassifier [77]	precision: 90 recall: 92 precision: 82 recall: 82 precision: 81 recall: 81 precision: 79 recall: 78 precision: 66 recall: 66 precision: 76 recall: 76 precision: 69 recall: 52 precision: 77 recall: 77
Control learning rate for autism facial detection via deep transfer Learning [34]	Deep Learning	Alexnet (84) Densnet121 [91] Mobilenet [89] Resnet18 [85] Sqeezenet [87] VGG11_ bn [83]	Average precision: 98 Average recall: 97
Comparing automated and nonautomated machine learning for autism spectrum disorders classification using facial images [35]	Machine Learning Deep Learning	LR ،LDA ،CART ،NB ،KNN ،SVM ،AB ،GBM ،RF ،ET CNN ،VGG16 ،VGG19 ،ResNet50 ،ResNet101 ،ResNet152	precision: 54, 56, 59, 67,67,78, 69,73, 69,76 NA
Autism spectrum disorder detection using facial images: A performance comparison of pretrained convolutional neural networks [21]	Deep Learning	ResNet34 [84] ResNet50 [87] VGG16 [81] VGG19 [87] AlexNet [85] MobileNetv2 [75]	Average precision: 93 Average recall: 88
Autism Spectrum Disorder detection in children by calculating the distances between facial landmarks [36]	Machine Learning	Logistic regression [82] K‐Nearest Neighbors [81] Random Forest [81] Decision tree [80] XGBoost [83]	precision: 79 recall: 89 precision: 82 recall: 79 precision: 81 recall: 82 precision: 81 recall: 78 precision: 84 recall: 83
A face image classification method of autistic children based on the two‐phase transfer learning [37]	Deep Learning	MobileNetV2 [88] MobileNetV3 [85]	Sensitivity: 86 Specificity: 90 Sensitivity: 91 Specificity: 83
A Methodology for Detecting ASD from Facial Images Efficiently Using Artificial Neural Networks [38]	Machine Learning	Artificial neural networks: Face_15_features: Activation function:Relu: 70Selu: 75 Face_8_features: Activation function:Relu: 70Selu: 63	MSE: 0.15
Autism spectrum Disorder detection Using Face Features based on Deep Neural network [39]	Deep Learning	VGG16 [73] Xception [94]	Sensitivity: 78 Specificity: 80 Sensitivity: 89 Specificity: 95
Efficient Net‐based Transfer Learning Technique for Facial Autism Detection [40]	Deep Learning	EfficientNetB0 [85]	precision: 77 recall: 100

**Table 3:** Facial features.

facial Region	Feature	Description	Reference
Upper Face	Wide upper face	Children with ASD often have a broader upper facial region	[12, 33, 36, 40]
	Wide‐set eyes	The eyes are typically positioned farther apart than in typically developing children	[12, 29, 31, 36, 40]
	Larger forehead	ASD‐positive individuals frequently have larger foreheads, possibly due to increased brain size during early development	[29]
Middle Face	Shorter middle region	The area encompassing the cheeks and nose is often shorter in children with ASD	[12, 31]
Lower Face	Larger mouth	Children with ASD often have broader or wider mouths	[31, 36, 37]
	Shorter philtrum	Shorter distance between the base of the nose and the border of the upper lip	[37]
Measurement Differences	Reduced distances	Measurements show shorter distances connecting the glabella and nasion to the inner canthi in children with ASD	[37]

### 2.4 Evidence Synthesis

We explained the results obtained from the study qualitatively, providing a comprehensive understanding of the findings and their implications.

## 3 Result

### 3.1 Study Selection

**Figure 1:**
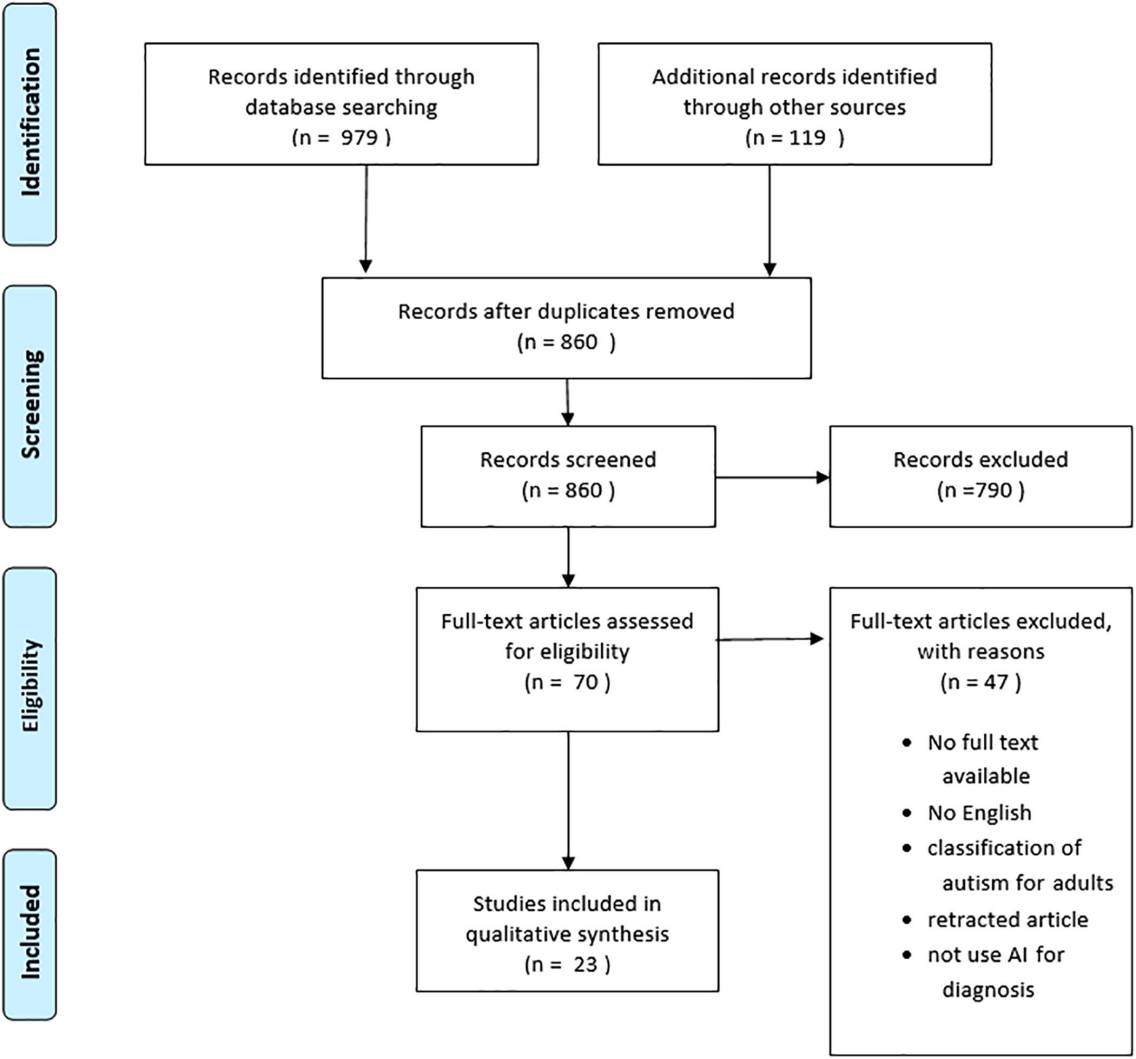
Flowchart for the selection process of the included studies.

The initial search strategy retrieved a total of 979 published articles without a time limit. After the removal of duplicate articles, 860 articles remained for screening. During the screening, 790 articles were discarded by title and 47 articles by full text. At the end of this process, 23 articles were included in this scoping review. Studies were identified in PubMed (*n* = 10), Scopus (*n* = 7), Web of Science (*n* = 3), and Scholar (*n* = 3). The flow of articles through identification to final inclusion is represented in Figure 1.

### 3.2 Characteristics of Studies

**Figure 2:**
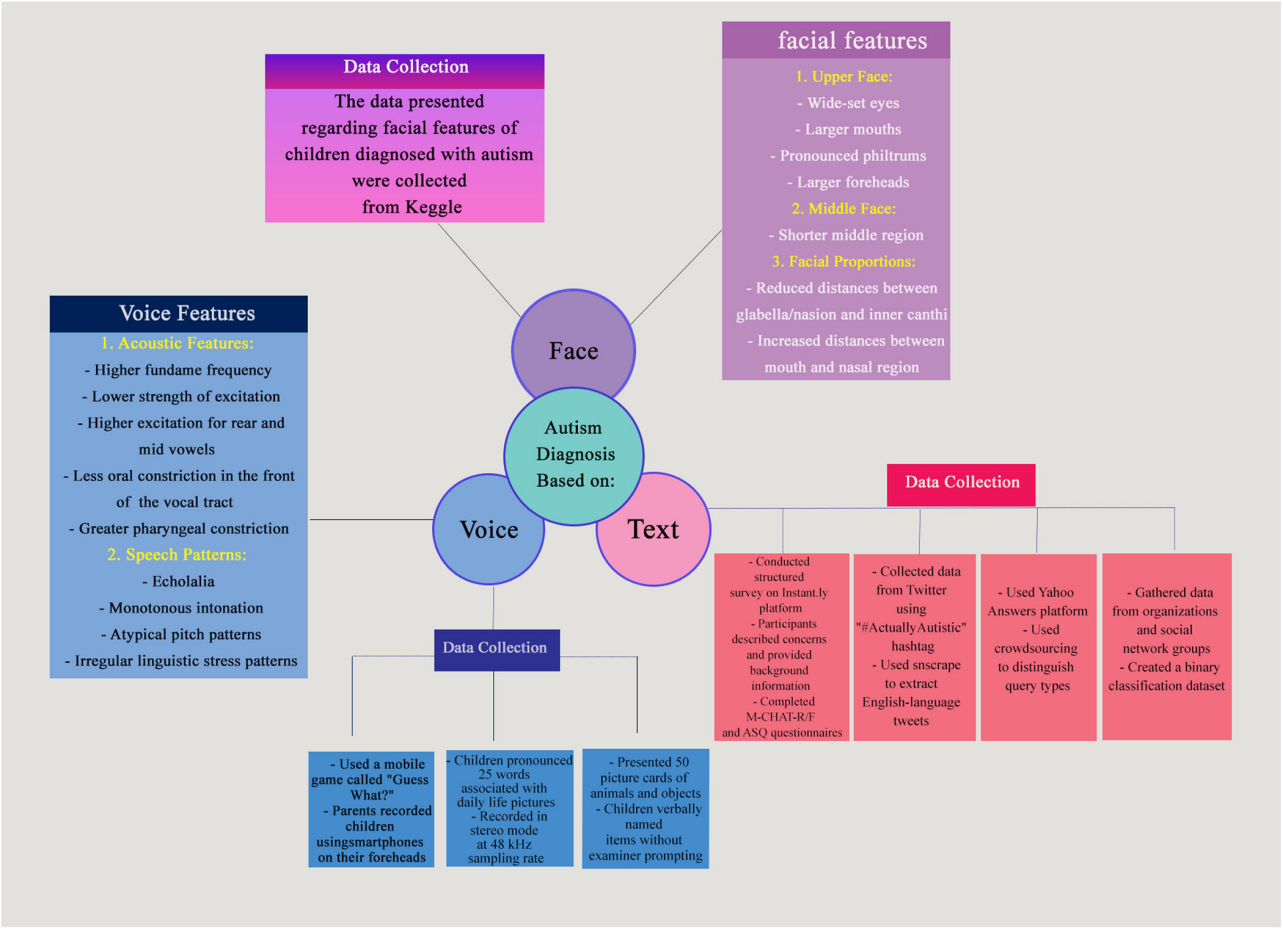
AI applications in autism diagnosis.

According to the included papers (see Supplementary Info 3), the main information is presented in the table, the diagnosis of children with autism into three areas: facial images, voice, and text. Each key component is described in detail, and related information is summarized in Figure 2.

### 3.3 AI Based Diagnosis of Autism through Facial Images

The data presented in this study regarding facial features of children diagnosed with autism spectrum disorder were collected from Kaggle [12, 21, 24, 26, 30, 31, 32, 33, 34, 35, 36, 37, 39, 40], utilizing an extensive data set comprising an average of 2900 photographs. This comprehensive collection allows for a robust analysis of the distinct facial markers associated with autism. Additionally, another study utilized the Autism Parenting Hub Facebook group [38] and external parties [29] as a source of data to gather relevant images.

Information about technical aspects is shown in Table 2:

The table provides a comprehensive overview of various algorithms used in deep learning and machine learning. Deep learning algorithms, particularly Convolutional Neural Networks (CNNs) like MobileNet, EfficientNet, and Xception, have shown high accuracy rates ranging from 85 to 98 out of 100 cases, indicating the quality or state of being correct. Traditional machine learning algorithms, such as Logistic Regression, Decision Trees, and Support Vector Machines, demonstrated moderate to good performance with accuracies between 62 and 82 out of 100 cases. Notably, hybrid and advanced approaches that combine elements of both deep learning and machine learning, such as XGBoost integrated with CNNs, achieved the highest accuracies, reaching up to 99%. The analysis of the results indicates that deep learning algorithms were employed across the majority of studies.

The features related to the faces of children with autism are shown in Table 3.

### 3.4 Standards for Facial Image Quality in ASD Diagnosis

Standards for the facial images included proper alignment of the images to accurately represent facial features, high image quality to avoid issues such as low brightness, small size, or blurriness, and ensuring that the entire face is visible rather than just one side [12].

### 3.5 AI Based Diagnosis of Autism Through Voice

The reviewed studies employed various methods for collecting speech data to diagnose children with Autism Spectrum Disorder through voice‐based analysis. One study utilized a mobile game called “Guess What?” which encouraged interaction between children and their parents, allowing parents to record their children's speech by positioning smartphones on their foreheads [41]. Another study adopted a structured approach, where verbal children aged 36 months and older were instructed to pronounce 25 specific words associated with daily life pictures, with recordings captured using a Roland R‐26 portable recorder [27]. The third study involved an experimental design in which participants named items from 50 picture cards without prompts from the examiner, aiming to assess spontaneous speech production [42].

Information about technical aspects is shown in Table 4:

**Table 4:** Technical aspects for voice.

Title (reference)	Data collection	Learning approach	AI algorithm (accuracy)
Classifying Autism from Crowdsourced Semistructured Speech Recordings: Machine Learning Model Comparison Study [41]	Mobile game “Guess What?” Recording Device: Smartphone front facing camera	Machine learning Deep learning Deep learning	Random Forest (69) CNN Model (79) Wav2vec 2.0 (76)
Analysis and classification of speech sounds of children with autism spectrum disorder using acoustic features [27]	Structured word pronunciation Recording Device: Roland R‐26 6‐channel portable recorder	Machine learning	Probabilistic neural network (98) Multilayer perceptron (98) Support vector machine (97) K‐nearest neighbors (96)
Detecting Abnormal Word Utterances in Children with Autism Spectrum Disorders: Machine‐Learning‐Based Voice Analysis Versus Speech Therapists [42]	Picture naming task	Machine learning	Machine‐learning based superior (76)

The table provides a comprehensive overview of various algorithms used in voice analysis for diagnosing Autism. Machine learning algorithms, such as probabilistic neural network (PNN) and multilayer perceptron (MLP), showed good performance with 98% accuracy. The analysis indicates a preference for machine learning approaches in the majority of studies, reflecting their effectiveness in processing voice data for ASD diagnosis.

The features related to the voice of children with autism are shown in Table 5.

**Table 5:** Voice Features.

**Feature**	**Description**	**Reference**
Contrastive stress	Differences in use, affecting clarity and emotional expression	[41, 42]
Emotional tone	Altered in speech	[42]
Pitch patterns	Irregular	[41, 42]
Fundamental frequency (F0)	Higher across all English vowels	[27]
Strength of excitation (SoE)	Lower for front vowels	[27]
Intonation	Monotonous (lack of pitch variation)	[41]
Vocal tract constriction	Less oral constriction in the front	[27]
Echolalia	Repetition of phrases or sentences	[41]

### 3.6 AI Based Diagnosis of Autism Through Text

The data collection methods for autism spectrum disorder research varied across four studies, each employing unique approaches to gather relevant information. One study collected data from organizations working with autistic children and social network groups, creating a binary classification data set that included dialogs from parents [19]. Another extracted 8681 autism‐related queries from Yahoo Answers, spanning 2006 to 2013 [6]. A third study conducted a structured survey on the Instant.ly platform, recruiting parents of young children with social‐communication concerns via social media [43]. The fourth study utilized Twitter, collecting over 3 million tweets with the hashtag “#ActuallyAutistic” from 2014 to 2022, creating a data set of self‐reported individuals with autism and a control group [44].

Information about technical aspects is shown in Table 6:

**Table 6:** Technical aspects for text analysis.

Title (Reference)	Data Collection	Learning approach	AI algorithm (accuracy)
Online concerns of parents suspecting autism spectrum disorder in their child: content analysis of signs and automated prediction of risk [6]	Yahoo Answers platform	Machine learning	Decision tree Distinguishing high risk: Using text alone: 67% Using coded signs: 82% Differentiating low risk: Using text alone: 54%Using coded signs: 84%
Risk assessment for parents who suspect their child has autism spectrum disorder: Machine learning approach [43]	Instant.ly platform	Machine learning	Machine learning techniques (NA)
Using #ActuallyAutistic on Twitter for Precision Diagnosis of Autism Spectrum Disorder: Machine Learning Study [44]	Twitter	Machine learning	SVM [61] Naive Bayes [59] Logistic regression [63] XGBoost [62]
Detection of Autism Spectrum Disorder (ASD) from Natural Language Text using BERT and ChatGPT Models [19]	Organizations working with autistic children, social network groups	State‐of‐the‐art language models	ChatGPT Model (NA) BERT Model [83]

Text analysis in autism diagnosis has employed a diverse range of artificial intelligence algorithms across multiple studies. These include machine learning techniques for analyzing parental concerns and questionnaires, decision tree algorithms for predicting autism risk from online queries, and a combination of supervised (naive Bayes, logistic regression, XGBoost) and unsupervised (Top2Vec with word2vec) models for examining social media data. An innovative approach integrated state‐of‐the‐art language models (BERT, ChatGPT) for natural language processing, achieving 83% accuracy in ASD detection from text.

## 4 Discussion

The evaluation of AI‐based techniques for the diagnosis of autism by facial expressions, voice, and textual analysis leads to several insights. Application of artificial intelligence across these modalities shows a lot of possibilities in the improvement of accuracy, efficiency, and accessibility of autism spectrum disorder (ASD) detection.

Facial analysis studies have shown distinctive features in children with ASD, including upper, middle, and lower facial asymmetry [12, 29, 31, 33, 36, 37, 40]. The highest reported accuracy in facial analysis for ASD detection was 98.9%, achieved using a deep learning model [26]. The second‐highest accuracy was 99%, using hybrid and advanced approaches [24]. Among deep learning models, Xception and EfficientNet variants consistently showed higher accuracies compared to others [12, 26, 31, 39, 40]. For machine learning algorithms, like XGBoost and Random Forest, performed well when integrated with deep learning techniques [24]. While, in the study of S. Suganyadevi et al. (2022), conducted a review of deep learning in medical image analysis, and they found that ResNet18 had the highest accuracy among deep learning algorithms with an accuracy of 99.40% [45]. In another study, Mohammad Shafiul et al. (2023), with the aim of evaluating the effect of different modalities of facial images on ASD diagnosis using a deep Learning‐Based Neural Network, found that Xception is showing the highest accuracy with 93.7% test accuracy while evaluating on the test set [46]. However, it is important to emphasize that facial features cannot be considered as the direct diagnostic criteria, as they may vary across individuals and populations. One of the debatable aspects of facial analysis studies for ASD detection is the use of similar or pre‐existing datasets across multiple studies. Race and ethnicity can significantly affect the facial feature analysis for the identification of ASD, which requires further investigation. Facial features can vary diverse across different ethnic populations, and it's essential to ensure that AI models are trained on diverse datasets to avoid bias and maintain accuracy across all populations.

In the study on ASD diagnosis by AI methods, different evaluation measures have been used; the range is wide, and each showcases a different aspect of model effectiveness. Although mostly a mixture of precision, recall, and accuracy has been reported by a great majority of papers, some other papers choose other approaches. For example, one paper did not report accuracy at all; rather, they focus on sensitivity (true positive rate), which measures how well the model identifies children with autism correctly, a factor peculiar to early diagnosis and intervention, and specificity (true negative rate) to say how well it identifies typically developing children, thus minimizing any false positives and unnecessary interventions [31].

Another paper evaluated most of its algorithms using recall and precision, metrics that represent the proportion of true positives correctly identified and the proportion of positive identifications that were actually correct, respectively. However, for one particular algorithm (in this study, whose accuracy was significantly lower), only AUC was reported [29]. This selective reporting likely reflects the algorithm's lower and more unstable performance and led the authors to highlight only those metrics where the results seemed more favorable.

Healthcare AI suffers from the great drawbacks and high risks of depending on a single data set. Bias is introduced in a variety of ways from collection to aggregation, all the way up to model interpretation. Should the data set not reasonably represent the rest of the population or contain a bias in history, these biased AI models may go one further by reinforcing existing inequalities in healthcare. This was the case with a recent case in which an algorithm underestimated the healthcare needs of black patients, using healthcare costs as a proxy for illness severity; a proxy already laden with bias due to the extant inequalities in healthcare access and resource allocation. However, even if the datasets were representative and measured correctly, they would mirror social inequalities at a deeper level. This type of historical bias cannot be easily corrected by adding more data, as it is rooted in real‐world injustices that are reflected in the data itself. Furthermore, medical research trends often prioritize the needs of more privileged populations, leading to data shortages and research focused on historically marginalized groups. Another important issue is overfitting. If a training set is biased, the model that the AI develops will overfit to the biased data and will not be able to handle other data well because it has never been exposed to it [47]. Aside from the above‐discussed points, it is important to consider that the image data on Kaggle does not specify under what standards the images were captured or collected. There is no information on procedures followed while imaging, equipment used, or the conditions under which images were taken. So, without any standardization for data capture, it becomes difficult to establish the reproducibility of results or the clinical applicability of the AI models developed using this data.

It is also important to note that, even if AI models perform well in research settings, they are not usually able to be utilized in real‐time clinical practice: this is due to variance in patient populations or differences in data gathering processes. Conversely, deep learning models may be less interpretable and thus less likely to be trusted and accepted by physicians. Also, these models with minuscule overfitting or generalization concerns can be trained on very few data, and most remain to be checked outside research settings, which questions their dependability for clinical purposes.

Voice analysis is quite effective, with some studies reporting accuracy rates as high as 98% using techniques such as probabilistic neural networks and multilayer perceptrons [27]. Among the deep learning approaches, the CNN model showed the best performance with 79% accuracy, while Wav2vec 2.0 achieved 76% accuracy [41]. Identifying specific vocal characteristics, such as atypical prosody, irregular pitch patterns, and differences in vowel pronunciation, provides a foundation for developing AI‐based voice analysis tools for ASD detection [27, 41, 42]. These methods could be valuable in assessing verbal children with ASD, as they can be applied non‐invasively to analyze speech patterns. However, it's necessary to state that vocal characteristics only emerge when a child can speak, which may be delayed or impaired in some children with ASD. It's worth highlighting that there isn't much research in this field of studying voice samples for ASD identification yet. While these findings are promising, more extensive studies with larger and more diverse samples are needed to validate these findings and explore their applicability across different age groups and severity levels of ASD.

Recent applications of text analysis supported by natural language processing models such as BERT and ChatGPT showed remarkable success in detecting linguistic markers of ASD. The reported accuracy of 83% in one study suggests that analyzing written text could provide valuable information on the communication patterns characteristic of ASD [19]. The use of text analysis for ASD diagnosis based on parent‐reported concerns has several advantages, including early detection potential, cost‐effectiveness, and complementarity to existing tools. This method can complement existing traditional assessments by providing additional information on parental concerns that may not be highlighted through standard diagnostic tools (Text analysis allows parents to express their observations and worries in their own words). It can be used as a pre‐screening tool, identifying children who may require further evaluation and enabling continuous monitoring of developmental changes. However, this method also has some problems, such as dependence on parental reporting, the possibility of bias, and variability in language use.

A notable limitation of the reviewed NLP studies was the inconsistent reporting of performance metrics. While some studies provided comprehensive measures such as precision, accuracy, recall, and F1_scores others relied on qualitative assessments or reported correlations without adequate benchmarking. For example, one study evaluated its machine learning models using the area under the receiver operating characteristic curve (AUC), with reported values ranging from 0.74 to 0.88 depending on the specific risk outcome. These findings suggest that in this particular study, the models demonstrated moderate to high levels of accuracy in discriminating between different risk categories [43]. However, the lack of additional measures, such as precision or recall, limits a more complete understanding of model performance, especially in unbalanced datasets. Across studies, the measurement and reporting of performance used to be inconsistent, and this hindered efforts towards comparing one study to another and judging the clinical utility.

The integration of AI‐based diagnostic tools has the potential to significantly increase screening and early intervention for autism spectrum disorder, particularly in areas with limited access to specialists. AI mobile applications and telehealth platforms might be considered complementary approaches to mass screening and triage‐aiding in the early detection and referral of children at risk. However, very few AI‐based diagnosis or screening tools find their way into everyday practice. Among other things, a few key challenges hamper the translation of research findings into practice: external validation is still necessary; regulatory approvals; physicians need to be educated; the recommendations from the AI must be explainable and trusted by the healthcare provider. Consequently, the study itself will most likely remain in experimental or research settings for years to come if these barriers remain unresolved.

While AI has a high potential in ASD detection, this approach should be considered as a complementary tool to support, not replace, human clinical judgment. The complex nature of ASD requires a general approach that considers not only biological markers but also social, environmental, and developmental factors that AI may not fully capture.

### 4.1 Limitation

A significant limitation of our study was the lack of a multilingual team to review all articles, which may have resulted in the exclusion of relevant non‐English publications. This linguistic constraint, coupled with the fact that the studies included might not represent a wide range of ethnic, cultural, or socioeconomic backgrounds, potentially limits the applicability of our findings to diverse populations. Additionally, our review considered an AI tool for ASD diagnosis only in the facial expression, vocal, and textual data, while all the AI techniques were applied exclusively for the detection of ASD diagnosis. Although these regions demonstrated promising diagnostic value, other major data modalities, such as neuroimaging (e.g., MRI, fMRI), electrophysiological data (e.g., EEG, MEG), and postmortem histology, were not included in the field of this review. These domains provide nuanced, interrelated perspectives on the neural and biological bases of ASD. Identified additional modalities should be incorporated into future reviews and meta‐analyses to offer a comprehensive and more balanced view on adjunct therapies.

## 5 Conclusion and Future Work

This review shows that it is possible for artificial intelligence to have a major, useful impact on the diagnosis of ASD. By synthesizing the findings from the present review, the emerging results have shown great promise in applying AI techniques in the early detection and diagnosis of ASD. Our study is based on the finding that many AI techniques have demonstrated a high level of accuracy for the detection of ASD markers. These algorithms have been used to analyze faces for a variety of purposes successfully. The results suggest that AI‐driven approaches can complement traditional diagnostic methods, potentially leading to earlier interventions and improved outcomes for individuals with ASD. The findings imply that AI‐enabled approaches can augment the traditional methods of diagnosis and thus contribute to earlier intervention and better intervention outcomes for individuals with ASD.

Given the fast development pace of AI, further studies may consider the use of advanced AI models, especially large language models (LLMs) such as BERT, GPT or their clinical versions to analyze linguistic and textual data. Such models can capture the fine‐grained semantic, syntactic, and pragmatic nature of the language (that could perhaps be considered as manifestations of ASD‐related language patterns). And the combination of them may bring some improvement in the sensitivity and specificity of the AI‐based evaluation on the text level.

Finally, research should also widen the range of types of AI models that are used on all existing data domains—not merely deep learning architectures for face‐based information, but newer and hybrid models for voice and language data as well. This more inclusive approach may lead to new discoveries and better generalization of diagnosis across ages, languages and cultures. These multimodal AI models may present a more nuanced, context‐based representation of social communication and behavior relevant to ASD. The next step for research would be to focus on building and testing such systems in different real‐world contexts. While, as we identified, it mostly focused on the facial image analysis task in ASD diagnosis, there are still a lot of possibilities to explore the voice and text analysis.

## Author Contributions

**Fatemeh Mohammadi:** conceptualization, data curation, formal analysis, investigation, methodology, resources, supervision, visualization, validation, writing – original draft, writing – review and editing. **Hassan Shahrokhi:** conceptualization, data curation, formal analysis, investigation, methodology, project administration, supervision, validation, writing – review and editing. **Afsoon Asadzadeh:** conceptualization, data curation, formal analysis, investigation, methodology, resources, supervision, validation, writing – review and editing. **Saeed Pirmoradi:** conceptualization, data curation, formal analysis, investigation, methodology, validation, writing – review and editing. **Ali Moghtader:** writing – review and editing, validation, formal analysis. **Peyman Rezaei‐Hachesu:** conceptualization, data curation, formal analysis, investigation, methodology, project administration, resources, supervision, validation, writing – review and editing.

## Conflicts of Interest

The authors declare no conflicts of interest.

## Supporting information


**S1 Table:** Preferred Reporting Items for Systematic reviews and Meta‐Analyses extension for Scoping Reviews (PRISMA‐ScR) Checklist.

**S2 Table:** Search Strategy in PubMed.

SuppInfo 3.

## Data Availability

The datasets used and/or analysed during the current study are available from the corresponding author on reasonable request.
