# Supplementary material for: Artificial Intelligence in Autism Spectrum Disorder Diagnosis: A Scoping Review of Face, Voice, and Text Analysis Methods
Source: Health Sci Rep. 2025 Nov 17;8(11):e71476. doi: 10.1002/hsr2.71476 (PMC12620671; doi:10.1002/hsr2.71476)
Supplement: Supplementary file 2 — S2 Table: Search Strategy in PubMed. [file HSR2-8-e71476-s002.docx]

| **ID** | **Details** | **Results** |
| --- | --- | --- |
| 1 | "Autistic Disorder"[Mesh] OR "Autism Spectrum Disorder"[Mesh] | 44,203 |
| 2 | (((((((((Autism spectrum disorder*[Title/Abstract]) OR (ASD[Title/Abstract])) OR (Autism[Title/Abstract])) OR (Autistic[Title/Abstract])) OR (autistic disorder*[Title/Abstract])) OR (Asperger syndrome*[Title/Abstract])) OR (Asperger*[Title/Abstract])) OR (pervasive developmental disorder*[Title/Abstract])) OR (high function*[Title/Abstract])) OR (high-function[Title/Abstract]) | 84,937 |
| 3 | 1 OR 2 | 88,050 |
| 4 | (((((("Artificial Intelligence"[Mesh]) OR "Machine Learning"[Mesh]) OR "Unsupervised Machine Learning"[Mesh]) OR "Supervised Machine Learning"[Mesh]) OR "Deep Learning"[Mesh]) OR "Neural Networks Computer"[Mesh]) OR "Natural Language Processing"[Mesh] | 189,198 |
| 5 | ((((((((((Artificial Intelligence[Title/Abstract]) OR (Machine Learning[Title/Abstract])) OR (Deep Learning[Title/Abstract])) OR (Neural Network*[Title/Abstract])) OR (Natural Language Processing[Title/Abstract])) OR (Computational Intelligence[Title/Abstract])) OR (Machine Intelligence[Title/Abstract])) OR (chat bot*[Title/Abstract])) OR (intelligent agent*[Title/Abstract])) OR (expert system*[Title/Abstract])) OR (AI[Title/Abstract])) OR (Computer Vision System*[Title/Abstract]) | 292,817 |
| 6 | 4 OR 5 | 371,262 |
| 7 | "Diagnosis"[Mesh] OR "Early Diagnosis"[Mesh] | 9,481,697 |
| 8 | (((((Diagnosis[Title/Abstract]) OR (Detect*[Title/Abstract])) OR (Screen*[Title/Abstract])) OR (identif*[Title/Abstract])) OR (predict[Title/Abstract])) OR (suspect[Title/Abstract]) | 8,647,000 |
| 9 | 7 OR 8 | 15,229,087 |
| 10 | "Voice"[Mesh]) OR "Voice Recognition"[Mesh]) OR "Voice Disorders"[Mesh] | 22,705 |
| 11 | "Speech"[Mesh]) OR "Speech Sound Disorder"[Mesh]) OR "Verbal Behavior"[Mesh] | 55,923 |
| 12 | (Voice[Title/Abstract]) OR (Voice Recognition[Title/Abstract])) OR (Voice Disorder*[Title/Abstract])) OR (audio[Title/Abstract])) OR (vocal[Title/Abstract])) OR (sound[Title/Abstract])) OR (Utterance[Title/Abstract])) OR (expression[Title/Abstract])) OR (speech[Title/Abstract])) OR (talk*[Title/Abstract])) OR (verbal[Title/Abstract]) | 2,874 |
| 13 | 10 OR 11 OR 12 | 2,896 |
| 14 | (Text*[Title/Abstract]) OR (Content[Title/Abstract])) OR (Content analysis[Title/Abstract])) OR (Narrative text[Title/Abstract]) | 1,083,900 |
| 15 | ((Parent Perceptions*[Title/Abstract]) OR (Parents Suspecting*[Title/Abstract])) OR (Concerns of Parents[Title/Abstract]) "parent perceptions*"[Title/Abstract] OR (("parent s"[All Fields] OR "parentally"[All Fields] OR "parentals"[All Fields] OR "parented"[All Fields] OR "parenting"[MeSH Terms] OR "parenting"[All Fields] OR "Parents"[MeSH Terms] OR ""Parents""[All Fields] OR ""Parent""[All Fields] OR "parental"[All Fields]) AND "suspecting*""[Title/Abstract]) OR ((""concern"[All Fields] OR "concerned"[All Fields] OR "concerning"[All Fields] OR "concerns"[All Fields]) AND ""of parents""[Title/Abstract]) | 4,111 |
| 16 | 14 OR 15 | 1,087,652 |
| 17 | "Facial Expression"[Mesh] OR "Facial Recognition"[Mesh] | 20,384 |
| 18 | "Face"[Mesh] | 175,894 |
| 19 | (Facial Expression[Title/Abstract]) OR (face recogn*[Title/Abstract])) OR (facial recogn*[Title/Abstract])) OR (face detect*[Title/Abstract])) OR (facial detect*[Title/Abstract])) OR (face photograph*[Title/Abstract])) OR (facial photograph*[Title/Abstract])) OR (face image*[Title/Abstract])) OR (facial image*[Title/Abstract])) OR (face analys*[Title/Abstract])) OR (facial analys*[Title/Abstract])) OR (face phenotype*[Title/Abstract])) OR (facial phenotype*[Title/Abstract])) OR (face feature*[Title/Abstract])) OR (facial feature*[Title/Abstract])) OR (face[Title/Abstract])) OR (facial[Title/Abstract]) | 391,292 |
| 20 | 17 OR 18 OR 19 | 530,846 |
| 21 | 13 OR 16 OR 20 | 4,339,535 |
| 22 | 3 AND 6 AND 9 AND 21 | 386 |

S2 Table: Search Strategy in PubMed
