# Supplementary material for: Artificial Intelligence in Autism Spectrum Disorder Diagnosis: A Scoping Review of Face, Voice, and Text Analysis Methods
Source: Health Sci Rep. 2025 Nov 17;8(11):e71476. doi: 10.1002/hsr2.71476 (PMC12620671; doi:10.1002/hsr2.71476)
Supplement: Supplementary file 3 — SuppInfo 3. [file HSR2-8-e71476-s001.docx]

| **author & year** | **Title** | **Country** | **Importance of Work** | **Sample size** | **Age** | **statistical analysis OR Validation** | **Main Results** | **Authors Conclusions** | **Users** |
| --- | --- | --- | --- | --- | --- | --- | --- | --- | --- |
| Israr Ahmad et al  (2024) | Autism spectrum disorder detection using facial images: A performance comparison of pretrained convolutional neural networks | Finland | there are currently no detectable clinical tests for this syndrome, such as a blood test, making the diagnosis a challenging task | 2940 images (kaggle) | NA | accuracy  precision  recall  error rate | 1. The proposed ResNet50 model achieved the highest accuracy of 92% compared to other models  2. The ResNet50 model surpassed state-of-the-art models in terms of accuracy and computational cost. | Models with 50 and 34 layers achieved high accuracy rates compared to MobileNetV2.  Pre-trained networks show promise for ASD detection using facial images. | providers |
| Tania Akter et al  (2021) | Improved transfer-learning-based facial recognition framework to detect autistic children at an early stage | Saudi Arabia | facial recognition plays an important role in recognizing a person’s identity or emotional state and can be used to detect autism effectively | 2936 images (kaggle) | 2 - 12 years | accuracy  AUC  f-measure  g-mean  sensitivity  specificity  fall-out | the MobileNet-V1 model consistently outperforms other classifiers, including general classifiers, CNN models, and improved pre-trained models, in both the test and validation sets. | The proposed framework can play a significant role in early autism detection and can be as a useful tool for physicians and health-workers. | physicians |
| Mohammad Shafiul Alam et al  (2023) | Efficient Deep Learning-Based Data-Centric Approach for Autism Spectrum Disorder Diagnosis from Facial Images Using Explainable AI | Saudi Arabia | traditional methods depend on the skilled physicians and the precision of the information provided by patients’ attendants or the parents. Recent advances in artificial intelligence have prompted the desire to implement it in this advanced medical diagnosis system. | 3014 images (kaggle) | 2 - 14 years | Accuracy  AUC  Precision  recall | 1.The ResNet50V2 model performed the best for training with a 99.9% accuracy and 100% AUC value, while the Xception model ranked first in testing with 92.5% accuracy and 97.9% AUC  2. The Xception demonstrates the highest testing accuracy, 98.9%, with a 99.9% AUC. | we highlight the importance of carefully observing distinct facial characteristics, including the forehead, area between the eyes, nostrils, lips, and occasionally the cheeks, in children diagnosed with autism | physicians |
| Md Shafiul Alam et al  (2022) | Empirical Study of Autism Spectrum Disorder Diagnosis Using Facial Images by Improved Transfer Learning Approach | Malaysia | the patient’s parents or attendant cannot always give accurate data or fill out the questionnaire forms correctly. All these factors can influence the accuracy of interview-based ASD diagnosis | 2940 images (kaggle) | 2 - 10 years | accuracy  precision  recall  AUC | The Xception model performs the best in terms of accuracy 95% and AUC compared to the other four referenced transfer learning approaches and an initial accumulator value of 0.01, while ResNet50V2 and MobileNetV2 obtained 94% and 92% accuracy, respectively | implementing the proposed model on mobile devices as one of the feasible solutions will be one of our primary concerns in future research | doctors and practitioners |
| Hasan Alkahtani et al  (2023) | Deep Learning Algorithms to Identify Autism Spectrum Disorder in Children-Based Facial Landmarks | Saudi Arabia | autistic individuals have a specific facial feature that is not shared by children who do not have autism | 2940 images (kaggle) | 2 - 14 years | sensitivity  specificity  precision  recall  accuracy | proposed model achieved an accuracy of 92%. The accuracy of MobileNet’s results was evaluated, with true-positives for normal and pathological classes presented on the y-axis and false-positives indicated on the x-axis | computer vision solution will help to address the major causes of racial disparity in ASD diagnosis and screening methods. | clinicians |
| Bakri Awaji et al  (2023) | Hybrid Techniques of Facial Feature Image Analysis for Early Detection of Autism Spectrum Disorder Based on Combined CNN Features | Saudi Arabia | certain facial features are associated with autism these features are not apparent to the naked eye, but they are analyzed using computer algorithms | 2940 images (kaggle) | 2 - 12 years | AUC  Accuracy  Precision  Sensitivity  Specificity | 1.the hybrid technique combining VGG16-ResNet101 with XGBoost achieved an accuracy of 97.3%  2. other combinations like ResNet101-MobileNet with XGBoost and VGG16-MobileNet with XGBoost also demonstrated high accuracy | By accurately identifying subtle facial cues associated with ASD, clinicians and researchers can identify children at risk much earlier, enabling timely intervention and support | clinicians |
| Abdelkrim El Mouatasim  Mohamed Ikermane  (2023) | Control learning rate for autism facial detection via deep transfer learning | Morocco | Develop a new approach to control the learning rate during training with CSA that leads to faster convergence and improved accuracy compared to the baseline method. | 2936 images (kaggle) | NA | Precision  Recall  f 1-score  Accuracy | the DenseNet-121 model is the best performing model for this particular dataset and problem | Use of the CSA optimizer in conjunction with the DenseNet-121 model and regularization significantly improved the classification accuracy for the task of identifying ASD in facial images | clinicians |
| Basma Ramdan et al  (2022) | Comparing automated and non-automated machine learning for autism spectrum disorders classification using facial images | Egypt | The body expressions of the subject with autism play a vital role in reflecting his or her behavior and feelings | 2936 images (kaggle) | NA | Precision  f 1-score  Accuracy | Using traditional ML algorithms, the best model was ET with an accuracy of 72.64%. using VGG16 architecture applied with transfer learning achieved an accuracy of 89% compared with 84% | we can create an optimized model using the AutoML method, which identifies autism in children from facial images. | clinicians |
| A. KALAISELVI et al  (2021) | Detection of autism spectrum disorder using transfer learning | Tamil Nadu | methods are less reliable, and validation of these results are subjective as it arises from a different trainer, and varies with the culture. Due these limitations and considerable increase in the autistic rate | 1648 images (kaggle) | NA | Accuracy  Precision  Recall  f 1-score | The NASNetLarge has the highest accuracy of 87.5% because it consists of more than million neurons to train and considers more features compared to the other models considered here | among the four models the NASNetLarge and Inceptionv3 model output performs the other two models namely VGG19, Resnet50 in classifying the input as autistic or not, as well as in terms of accuracy and loss. | clinicians |
| T. Lakshmi Praveena  N. V. Muthu Lakshmi  (2020) | A Methodology for Detecting ASD from Facial Images Efficiently Using Artificial Neural Networks | India | ASD individuals have differences in facial landmarks points and coordinates. The changes of brain are reflected in face, such as “Face predicts the brain” | 50 ASD images collected from Autism Parenting Hub face book group  50 images of TD group | 1 - 10 years | Accuracy  R square  Mean | The main land The document outlines the use of ANN for processing facial images efficiently, and the significance of early detection for improving treatment outcomes for ASD individuals and ANN demonstrates the highest accuracy | ANN plays an important role in prediction, detection and classification of data. ANN gives very good results in disease prediction and detection in health care | clinicians |
| Ying Li et al  (2023) | A face image classification method of autistic children based on the two-phase transfer learning | United States | With the progress and popularization of mobile technology, it is convenient to use a mobile phone to photograph and analyze children’s faces, and then draw the conclusion whether the child is autistic | 2340 images (kaggle) | NA | Accuracy AUC Error_rate Sensitivity Specificity G_Mean F_Measure | the effect of the two-phase transfer learning of MobileNetV2 is better than that of the one-phase transfer learning.  After the two-phase transfer learning, the sensitivity is significantly improved (5.07%), while the specificity is slightly improved (0.05%) | The differences between this research and the existing studies lie in the different transfer learning methods and size of an input image (224 × 224 or 299 × 299) | clinicians |
| Jyoti Madake et al  (2023) | Autism Spectrum Disorder detection in children by calculating the distances between facial landmarks | India | Despite the fact that the disease is thought to be genetic, testing a kid for behavioral traits and facial features results in the highest chances of accurate diagnosis | 2726 images (kaggle) | NA | Accuracy  Precision  Recall  F1 Score  F2 Score | It was observed that XGBoost gave the highest accuracy of 83.5%. the accuracy of Logistic Regression was 82.8%  For KNN, the accuracy was 81.5%. The Random Forest also gave an accuracy of 81.5% .The accuracy of decision tree was 80.1% after setting max_features. | The use of Haar cascade for the detection of facial landmarks makes this model unique because it utilizes machine learning and computer vision techniques rather than deep learning and CNN | clinicians |
| TARIQ SAEED MIAN  (2023) | EFFICIENT NET-BASED TRANSFER LEARNING TECHNIQUE FOR FACIAL AUTISM DETECTION | Saudi Arabia | Diagnostic methods are expensive and out of reach, especially in developing countries | 2536 images (kaggle) | NA | Accuracy  Precision  Recall  F1 Measure | Training loss gradually decreases, and the problem of overfitting is resolved  We identified the model’s bias toward non-autistic class cases in terms of accuracy. We also evaluate the effectiveness of the suggested method in terms of recall and precision and F1-score | To address the issue of transfer learning, we presented an approach by using last three layers of EfficientNetB0 for detecting autism disorder as not having enough training data. | clinicians |
| K. K. Mujeeb Rahman  M. Monica Subashini  (2022) | Identification of Autism in Children Using Static Facial Features and Deep Neural Networks | India United Arab Emirates | facial dysmorphologies, or aberrant facial traits induced by abnormalities in the embryonic development process, are strongly linked to the underlying neurological issues | 2936 images (kaggle) | 2 - 14 years | specificity  sensitivity  NPV  PPV  AUC | the Xception model outperformed the MobileNet model across the board, with scores of 88.46% sensitivity, 91.66% specificity , 88.00% NPV, 92.00% PPV, and AUC of 96.63% | The findings show that the distinct features of ASD can be efficiently gathered from static face images of a child | clinicians |
| Lubnaa Abdur Rahman  Poolan Marikannan Booma  (2022) | The Early Detection of Autism Within Children Through Facial Recognition; A Deep Transfer Learning Approach | Malaysia | late diagnosis also occurs from the fact that parents, themselves, are not aware that their children have a developmental disorder due to mainly knowledge gap. Therefore, using facial features of their kids, they could potentially identify probable autism as early as possible | 2936 images  ( external parties) | NA | Accuracy  Precision  Recall  F1 Measure | the MobileNet model trained was the one with the highest performance based on accuracy, loss and AUC values and is said to be the best fit for this problem as opposed to VGG16 and ResNet50. | MobileNet proved to be the most performant model with the highest accuracy of 87.5% and high AUC of 0.93 reached while, as opposed to previous works, ResNet50 failed to deliver adequate performance | clinicians |
| Ali F. Rashid  Shaimaa.h. shaker  (2023) | Autism spectrum Disorder detection Using Face Features based on Deep Neural network | Iraq | Facial recognition technology is frequently used to recognize people and establish whether they are normal or aberrant. In order to identify behavioral patterns, it includes mining relevant information | 2940 images (kaggle) | NA | Accuracy  Sensitivity  Specificity | They demonstrate that the VGG16 model had the lowest performance level at 78% and Xception model had the greatest accuracy of testing, 91% | The model’s classification results have shown the possibility of the use of such DL and computer vision models as automated tools for the professionals and families to more quickly diagnose autism | Clinicians |

Table. Extraction Table: Facial images

| **author & year** | **Title** | **Country** | **Importance of Work** | **Sample size** | **Age** | **statistical analysis OR Validation** | **Main Results** | **Authors Conclusions** | **Users** |
| --- | --- | --- | --- | --- | --- | --- | --- | --- | --- |
| Nathan A Chi et al (2022) | Classifying Autism From Crowdsourced Semistructured Speech Recordings: Machine Learning Model Comparison Study | United States | children display peculiarities including echolalia, monotonous intonation, and atypical pitch and linguistic stress patterns. Given this, it would be a valuable tool to aid autism diagnostic processes | total of 77 videos of 58 children | 2 - 8 years | Accuracy  Precision  Recall  F1 score  AUROCa | the best-performing model was the CNN model with 8 million parameters, achieving 79.3% accuracy, 80.4% precision, 79.3% recall, 79.0% F1 score, and a mean AUROC score of 0.822 | Use of automatic audio classification could help to accelerate and improve the accuracy and objectivity of the lengthy diagnosis process for autism | clinicians |
| Abhijit Mohanta Vinay Kumar Mittal (2022) | Analysis and classification of speech sounds of children with autism spectrum disorder using acoustic features | India | a noteworthy delay and repetition of spoken language can be observed among many individuals with ASD | 33 children | less than 36 month | AFCs  Accuracy  F1-score  p-value | *The study achieved a high classification accuracy rate of 98.17% using ML classifiers  *Children with ASD were found to have lesser lip-rounding during the vocalization of all English vowels compared to Normal children | The acoustic characteristics of the children with ASD observed in this study may be utilized as an acoustic biomarker to diagnose ASD, especially from Indian children’s speech sounds, at a very early age | clinicians |
| Yasushi Nakai et al (2017) | Detecting Abnormal Word Utterances in Children With Autism Spectrum Disorders: Machine-Learning-Based Voice Analysis Versus Speech Therapists | Japan | Abnormal prosody in individuals with ASD persists, even when other features improve, and it can be a significant social barrier | 30 children with ASD 51 children considered TD | 3 - 10 years | F-measure  Accuracy  True positive rate  False negative rate  False positive rate  True negative rate | The F-measure was significantly higher with ML based voice analysis than with speech therapist judgments, whereas accuracy was similar between machine-learning-based voice analysis and speech therapist judgments | NA | clinicians |

Table. Extraction Table: Voice

| **author & year** | **Title** | **Country** | **Importance of Work** | **Sample size** | **Age** | **statistical analysis OR Validation** | **Main Results** | **Authors Conclusions** | **Users** |
| --- | --- | --- | --- | --- | --- | --- | --- | --- | --- |
| Ayelet Ben Sasson Elad Yom Tov (2016) | Risk Assessment for Parents Who Suspect Their Child Has Autism Spectrum Disorder: Machine Learning Approach | Israel | many parents of pediatric patients use the internet to research medical information and relieve anxiety, they are likely to find incorrect advice, and only 21% share this information with their health care provider | 115 parents | 16 - 30 months old | Spearman correlations  Chi-square tests  AUC | 1.Screening measures identified 58% to 88% of children at risk for autism spectrum disorder  2. Children with a family history of autism spectrum disorder were 3 times more likely to show autism risk on screening measures | This study offers an opportunity to capitalize on digital health methods for designing a real-time support system that would ask users screening questions based on their concerns. | parent |
| Aditi Jaiswal  Peter Washington  (2024) | Online Concerns of Parents Suspecting Autism Spectrum Disorder in Their Child: Content Analysis of Signs and Automated Prediction of Risk | Israel | parental concerns about behavioral problems and about cognitive delay in the absence of concerns about communication were not likely predictors of an ASD diagnosis. This evidence underscores the need for an automated system, which can capture the combination of concerns in risk determination. | A total of 195 queries | mean child age=38 months | 1.analysis of Yahoo Answers queries was conducted using the NVIVO software, two types of content analysis processes were applied:  a. content analysis  b. deductive and inductive analysis  2. Bonferroni | Concerns related to repetitive and restricted behaviors and interests (RRBI) were the most prevalent (75.4%, 147/195), followed by concerns related to language (61.5%, 120/195) and emotional markers (50.3%, 98/195) | This study’s findings support the call for health care providers to closely listen to parental ASD-related concerns, as recommended by screening guidelines | providers |
| Prasenjit Mukherjee et al (2023) | Using #ActuallyAutistic on Twitter for Precision Diagnosis of Autism Spectrum Disorder: Machine Learning Study | United States | nonclinical data hold considerable potential for clinicians and researchers to extract meaningful insights through a less intrusive approach | 6,515,470 tweets | NA | For text-to-numeric vectorization, we used 2 approaches: 1. a bag-of-words term frequency–inverse document frequency (TF-IDF) method and 2.word2vec embeddings | A 0.728 area under the receiver operating characteristic curve score, and an 0.71 F1-score using word2vec representations fed into a logistic regression model | Textual differences in social media communications can help researchers and clinicians conduct symptomatology studies in natural settings | researchers and clinicians |
| Ayelet Ben Sasson Elad Yom Tov (2016) | Detection of Autism Spectrum Disorder (ASD) from Natural Language Text using BERT and ChatGPT Models | India | Parents of autistic children are spent most of their time with their children and their experience is the key to detecting ASD symptoms perfectly | various social networks and organizations | NA | Precision  Recall | 1. the BERT model demonstrated the ability to detect sentiment related to ASD symptoms with an accuracy of 83%.  2. ChatGPT Model: was fine-tuned and pre-trained to achieve high accuracy in detecting ASD symptoms based on the sentiment expressed in the text. | The proposed system has the potential to facilitate ASD detection and support in underserved regions, thus bridging the gap in ASD diagnosis and intervention | providers |

Table. Extraction Table: Text Analysis
